# Acaricidal activity of *Laurus nobilis* L. against *Varroa destructor*: nanoemulsion and molecular docking

**DOI:** 10.1186/s12917-026-05669-4

**Published:** 2026-06-23

**Authors:** Mustafa Güneşdoğdu, Samet Hasan Abacı

**Affiliations:** 1 Department of Animal Production and Technologies, Faculty of Applied Sciences, Muş Alparslan University, Muş, 49250 Türkiye; 2 Department of Animal Science, Faculty of Agriculture, Ondokuz Mayıs University, Samsun, 55139 Türkiye

**Keywords:** Efficacy, Essential oil, Mite, Colony-level, AChE inhibition

## Abstract

**Background:**

*Varroa destructor* (Acari: Varroidae) is one of the most significant threats to honeybee health, and sustainable alternatives to synthetic acaricides are urgently needed. Essential oil (EO)-based nanoformulations have emerged as promising candidates due to their enhanced stability and bioavailability.

**Methods:**

In this study, the acaricidal effect of a nanoemulsion (NEms) of *Laurus nobilis* L. EO was evaluated through field trials. The chemical composition of the oil was analyzed by gas chromatography–mass spectrometry (GC-MS), and the physicochemical properties of the NEms were characterized. Additionally, molecular docking analysis was performed to investigate interactions between major components and acetylcholinesterase (AChE).

**Results:**

GC-MS analysis revealed 1,8-cineole (40.65%) as the main component, followed by α-terpinyl acetate and β-phellandrene. The NEms exhibited a mean droplet size of 72.36 nm with good stability. Field results demonstrated a dose-dependent reduction in mite density, reaching 70.3% efficacy at 200 ppm, compared to 40.2% for the positive control (flumethrin). Molecular docking showed strong binding interactions with AChE, with α-terpinene exhibiting the highest affinity (-6.874 kcal/mol), followed by 1,8-cineole (-6.163 kcal/mol).

**Conclusions:**

These findings indicate that *L. nobilis* NEms may represent an effective and environmentally friendly alternative for controlling *Varroa* mites, supported by a plausible neurotoxic mechanism. To our knowledge, this study is among the first to integrate NEms -based field efficacy with AChE-targeted molecular docking, linking biological activity with mechanistic insight.

**Supplementary Information:**

The online version contains supplementary material available at https://doi.org/10.1186/s12917-026-05669-4.

## Introduction

Honeybees (*Apis mellifera* L.) are essential for ecological balance and agricultural productivity. Nearly 70% of global flowering plants rely on bee pollination, making bees vital for food security and biodiversity [1]. However, honeybee populations face severe global threats [2]. The ectoparasite *Varroa destructor* is currently the most destructive factor driving colony losses [3]. This mite feeds directly on larval hemolymph [4] and adult fat body tissue [5]. Such feeding weakens bee immunity and accelerates the transmission of lethal viruses, including deformed wing virus, acute bee paralysis virus, and Kashmir bee virus [6]. These combined viral infections impair overall fitness, frequently causing sudden colony collapse [7]. Consequently, mitigating *Varroa* infestation remains a critical priority in apiculture.

Synthetic acaricides have long served as the primary control method. However, intensive chemical treatments trigger rapid resistance development, cause environmental toxicity, and leave harmful residues in bee products [8]. These setbacks drive the search for eco-friendly alternatives. Plant-derived essential oils (EOs) are promising biopesticides for *Varroa* control [9]. Some EOs show acaricidal toxicity comparable to synthetic chemicals [10], while others display moderate efficacy or strictly repellent properties [11]. Despite variable field outcomes, EOs remain highly attractive due to their natural origin and low toxicity towards non-target organisms [12]. Nevertheless, optimizing their safety margins and stable field efficacy requires rigorous evaluation [13].

Plants synthesise diverse secondary metabolites primarily for chemical defence [14]. Modern phytotherapy increasingly adopts these phytochemicals as safe antimicrobial [15] and antiparasitic agents [16]. *Laurus nobilis* L. (Lauraceae), or laurel, is a Mediterranean aromatic plant widely used in food and traditional veterinary medicine [17]. Laurel extracts exhibit strong antibacterial [18], insecticidal [19], and acaricidal properties [20], attracting significant scientific interest [21]. Chemically, laurel EO is rich in bioactive terpenes, primarily 1,8-cineole, α-terpinyl acetate, sabinene, linalool, and methyl eugenol [22], as well as α-pinene and β-pinene [9].

Despite their bioactivity, raw EOs face practical limitations due to high volatility and low chemical stability. Nanotechnology addresses these constraints through advanced delivery systems [23]. In agricultural pest management, nano-pesticides improve biological efficiency via increased surface area interactions [24]. Nanoemulsions (NEms) use nanoscale droplets to stably disperse immiscible oil phases in water [25]. This encapsulation prevents rapid volatilisation, improves chemical stability, and ensures controlled release, enhancing toxicity against target pests [26]. However, the application of EO-based NEms against *Varroa* mites remains an understudied area requiring precise formulation testing under diverse field conditions [27].

Targeting specific physiological pathways is essential for developing effective biopesticides. Acetylcholinesterase (AChE) is a key cholinergic enzyme that hydrolyses acetylcholine to terminate nerve impulse transmission at synapses [28, 29]. This vital component of the nervous system is also highly functional in arthropod pests, including mites [30]. In invertebrates, neuromuscular regulation relies heavily on AChE, making it a primary target for chemical control [31]. Disrupting AChE leads to toxic acetylcholine accumulation, resulting in continuous synaptic stimulation, paralysis, and death [32]. Therefore, identifying natural compounds that inhibit this enzyme is a major focus in integrated pest management [33–35].

While the gross acaricidal effects of laurel oil are known, the precise molecular interactions between its major chemical constituents and the targeted AChE remain unresolved. Due to the absence of a crystallised *Varroa destructor* AChE structure in the Protein Data Bank (PDB), the well-characterised crystal structure of *Drosophila melanogaster* AChE (PDB ID: 6XYY) was used as a reliable homologous model for arthropod cholinergic systems. In this study, the binding affinity and specific structural interaction profiles of GC-MS-identified *Laurus nobilis* constituents with this target were investigated using in silico molecular docking. Concurrently, to validate these findings at the apiary level, the actual acaricidal efficacy and colony tolerability of the developed nanoformulations were evaluated through field trials using three distinct concentrations.

The primary novelty of this research lies in its specific application methodology and formulation characteristics. NEms systems remain exceptionally scarce in apicultural pest management [36]. While recent baseline attempts have used nano-formulations via dietary candy incorporation [36], this study establishes the first framework evaluating *Laurus nobilis* nanoemulsions via direct topical spray application. By transitioning from oral feeding delivery to a targeted contact-based liquid spray, the nanoscale droplets are allowed direct interaction with both the host cuticle and the ectoparasite. By bridging computational molecular modelling with this novel real-world field application, a comprehensive framework is provided for utilising laurel-based NEms as targeted, residue-free alternatives in *Varroa* management.

## Materials and methods

### Study area and colony characteristics

This research was carried out in an open-air apiary owned by the corresponding author in Suvaran village, Muş Province, Türkiye (38.7714° N, 41.4306° E). The apiary comprised 25 Langstroth hives fitted with plastic bottom boards and pollen traps, each containing a single brood chamber with nine frames. The experimental colonies consisted of naturally mated Caucasian F1 hybrid queens that had been introduced in June [8]. These colonies exhibited adequate brood development, generally covering approximately two frames of sealed brood per hive. To ensure consistency among experimental units, colonies were standardized in terms of strength, population size, brood level, and food reserves [37]. No routine treatments were applied against *Varroa* infestation, allowing the mite population to develop under natural conditions. During September, the mean ambient temperature was recorded as 20.1 °C, and within the optimal range of 10–25 °C recommended for EO-based *Varroa* control [38]. The relative humidity was approximately 50%, which is below the threshold (< 60%) known to affect the survival of bees infested with *V. destructor* [39]. Meteorological data recorded throughout the study period are presented in Supplementary Table S1.

### Essential oil and chemical characterization

Laurel EO used in this study was obtained from a commercial supplier in İzmir, Türkiye. The oil was stored at + 4 °C under refrigerated conditions until used in subsequent analyses and preparation procedures. The chemical composition of the EO was analyzed according to the method described by Ligor et al. [40], with slight modifications. For this purpose, a gas chromatography–mass spectrometry (GC–MS) system (PerkinElmer Clarus 500, PerkinElmer Inc., Waltham, MA, USA), combining capillary gas chromatography with mass spectrometric detection, was employed. Separation of volatile compounds was achieved using a non-polar fused silica capillary column (SGE BPX5, 60 m × 0.25 mm inner diameter, 0.25 μm film thickness, USA). The oven temperature program was set to an initial temperature of 60 °C, maintained for 10 min, followed by a gradual increase at a rate of 4 °C/min to 250 °C, where it was held for an additional 10 min. The injector temperature was fixed at 220 °C. Helium served as the carrier gas at a constant flow rate of 1.5 mL/min. Prior to injection, the EO was diluted in hexane at a ratio of 1:100 (v/v), and 1 µL of the prepared solution was introduced into the system in splitless mode. Mass spectrometric detection was carried out using electron ionization at 70 eV. The ion source and interface temperatures were maintained at 200 °C and 250 °C, respectively. Mass spectra were recorded over a scan range of m/z 35–425. Identification of volatile constituents was performed by comparing the obtained mass spectra with those available in the NISTMS and WILEYMS 9 spectral libraries (National Institute of Standards and Technology, USA).

### Nanoemulsion formulation and preparation

**Fig. 1:**
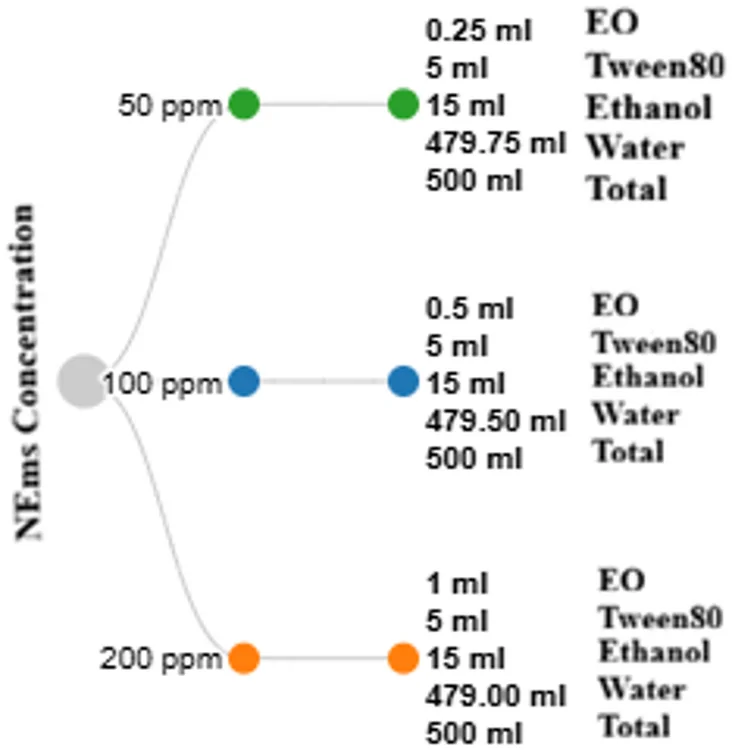
Preparation scheme and composition of nanoemulsion formulations (*NEms: nanoemulsion; 50, 100, 200 ppm: nanoemulsion concentrations; EO: essential oil)

NEms formulations (oil-in-water type) were prepared based on a modified protocol described by Joe et al. [41]. The composition of the different NEms formulations is presented in Fig. 1. In brief, the EO, Tween 80 (as a surfactant), and ethanol (as a co-surfactant) were initially mixed and heated at 86 °C for 1 h. Considering that the boiling point of ethanol is 78.4 °C, it was assumed to evaporate during this heating process [42]. Following this step, the mixture was diluted with double-distilled water to obtain a final volume of 500 mL. Prior to ultrasonic processing, the formulation was pre-homogenized using a mechanical homogenizer (IKA T25 digital ULTRA TURRAX) at 500 rpm for 15 min. Subsequently, the emulsion was subjected to ultrasonic homogenization for 15 min at 72% amplitude using an ultrasonic homogenizer (Optic Ivymen System CY-500, Barcelona, Spain), operating at 500 W and 20 kHz. A sonotrode with a diameter of 5.6 mm and a length of 60 mm was employed. During sonication, the temperature was maintained at approximately 15 °C using an ice bath. Ethanol was used as a co-surfactant in the NEms system.

### Physicochemical properties of NEms

The mean droplet size of the NEms was determined using a Zetasizer (Version 7.13; Malvern Instruments Pvt. Ltd., UK). The stability of the formulations was evaluated over a period of two weeks. For stability assessment, samples were subjected to centrifugation at 2000 × g for 30 min on alternate days at two different temperatures (4 °C and 45 °C). Following each centrifugation cycle, the samples were kept at room temperature (23–24 °C). At the end of the 14-day observation period, no signs of phase separation or formation of an oil layer were detected, indicating good physical stability of the NEms [42].

### Experimental design

The experimental concentrations of the EO were determined with slight modifications based on the values reported by Gamal Eldin et al. [36]. In this study, laurel EO was evaluated at three different concentrations (50, 100, and 200 ppm). The colonies were divided into three treatment groups receiving NEms formulations of EO (15 colonies in total), one positive control group treated with flumethrin (5 colonies), and one negative control group (5 colonies). Flumethrin (Bayvarol^®^), a widely used synthetic acaricide, was applied to the positive control colonies in accordance with standard beekeeping practices. EO applications were performed by spraying using a hand-held sprayer at a dose of 5 mL per frame, following the method described by Damiani et al. [43] with minor modifications. All treatments were administered five times at 7-day intervals (days 0, 7, 14, 21, and 28).

To accurately evaluate treatment efficacy, untreated control groups were included to distinguish between treatment-related mite mortality and natural reductions resulting from colony defense behaviors such as grooming and hygienic activity. The infestation level of *Varroa* mite in adult worker bees was determined as the number of mites per 10 g of bees, based on samples of approximately 100 worker bees [44]. The sugar roll method described by Seven-Çakmak et al. [45] was used for this purpose. Briefly, approximately 10 g of adult worker bees were placed in a glass jar fitted with a fine mesh lid, and 20 g of powdered sugar was added. The jar was gently shaken for 2 min to dislodge mites from the bees. Subsequently, the jar was inverted over a white surface, and the mites, along with the sugar, were collected and counted. The bees were then returned to their original colonies. *Varroa* infestation levels were recorded prior to each treatment and on days 0, 7, 14, 21, and 28. Treatment efficacy was calculated according to the method described by Güneşdoğdu and Abacı [46], using the following formulas:

1 $$\%Infestation\;(varroa\;per\;10g\;of\;bees)=\frac{Total\;number\;of\;dropped\;varroa\times10}{Net\;weight\;of\;bees}$$

2 $$\%\;Change\;in\;mite\;population=\left[\left(\frac{Initial\;varroa\;count-Final\;varroa\;count}{Initial\;varroa\;count}\right)\right]\times100$$

Additionally, fallen mites were monitored using Vaseline-coated white paper placed in pollen drawers, a commonly used and reliable method for mite monitoring [47]. Mite fall counts were conducted on days 1, 3, and 5 following each treatment across all experimental groups.

### Molecular docking study

Molecular docking is a widely used computational method in drug discovery and development to estimate the binding mode and interaction affinity of small molecules with specific biological targets [48–54]. The major phytochemical constituents of *Laurus nobilis* (laurel) EO were identified by GC-MS analysis, and the five most abundant compounds; eucalyptol (1,8-cineole), α-terpinyl acetate, β-phellandrene, 1R-α-pinene, and (-)-β-pinene, were selected for molecular docking studies. As there is currently no crystallised *Varroa destructor* AChE structure available in the PDB, the well-characterised crystal structure of *Drosophila melanogaster* AChE (PDB ID: 6XYY) was retrieved from the PDB as a reliable homologous model for arthropod cholinergic systems [31]. Before docking, the protein structure was prepared using AutoDock Tools (ADT, version 1.5.7). Preparation involved removing crystallographic water molecules, adding polar hydrogen atoms, and assigning Gasteiger partial charges. The prepared protein structure was then saved in PDBQT format. Ligand structures were obtained from the PubChem database in SDF format and converted to PDB format using BIOVIA Discovery Studio Visualizer 2025. Ligands were further prepared by assigning appropriate torsions and performing energy minimization where necessary. Molecular docking calculations were performed using AutoDock Vina [55]. The docking grid was defined around the enzyme’s active site with the following parameters: grid spacing of 0.375 Å, grid size of 20 × 20 × 20 points, and grid center coordinates at x = 24.432, y = 63.257, and z = 10.018. For each ligand, multiple binding conformations were generated. The pose with the lowest binding free energy (ΔG) was selected as the most favorable binding mode. Protein-ligand interactions, including hydrogen bonding, hydrophobic interactions, and π-related interactions, were analyzed and visualized in both two-dimensional (2D) and three-dimensional (3D) formats using BIOVIA Discovery Studio Visualizer 2025.

### Statistical analysis

The normality of the variables examined in the study was assessed using the Shapiro-Wilk test. Results indicated that all variables were normally distributed (*p* > 0.05). A repeated-measures mixed-design ANOVA was subsequently applied to examine the variables of interest. Prior to the main analysis, the sphericity assumption was evaluated using Mauchly’s Test of Sphericity, which yielded a statistically significant result (*p* < 0.05), indicating a violation of the sphericity assumption. Therefore, degrees of freedom were corrected using the Greenhouse-Geisser epsilon (ε) correction, and the adjusted results were reported accordingly.

To identify the statistically significant differences among repeated measurements across time points or conditions, post hoc multiple-comparison analyses were conducted. The Bonferroni test was applied to control for the Type I error rate across multiple comparisons. Descriptive statistics are presented as mean ± standard error of the mean (SEM). Statistical analyses were carried out using SPSS software (SPSS Inc., Chicago, IL, USA), and graphical visualizations were generated using the SRplot online platform [56]. A statistical significance threshold of *p* < 0.05 was adopted for all analyses.

## Results

### GC-MS compounds of laurel essential oil

**Fig. 2:**
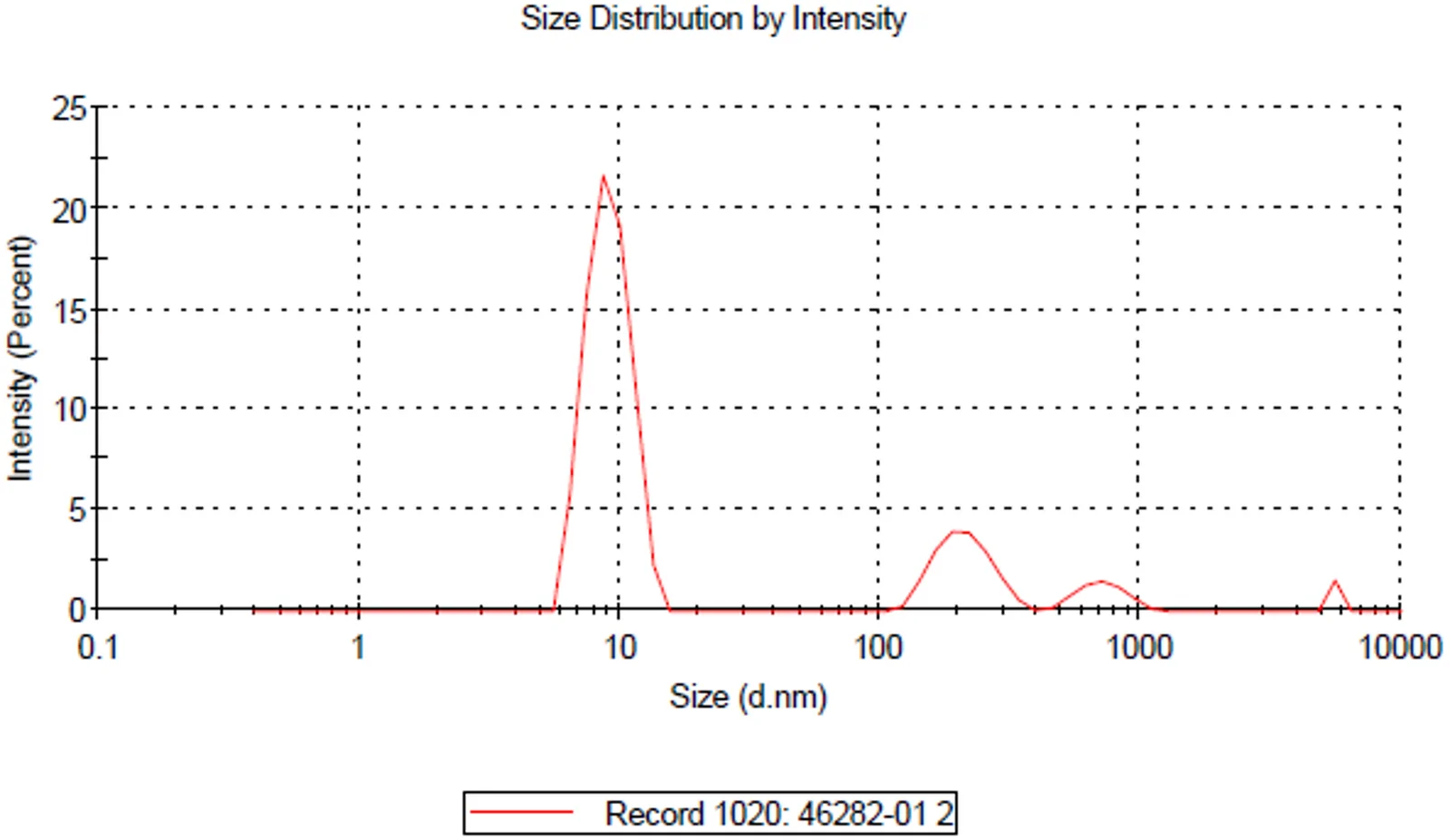
Particle size distribution of EO-based NEms

In this study, the chemical composition of Laurel (*Laurus nobilis* L.) EO was analyzed, and the percentage distribution of its components is shown in Table 1. The results indicate that the most abundant component of the EO is eucalyptol (1,8-cineole), accounting for 40.65%. This is followed by α-terpinyl acetate at 14.51%, β-phellandrene at 9.53%, 1R-α-pinene at 6.80%, and (-)-β-pinene at 5.46%. These five compounds constitute a significant proportion of the total composition and define the characteristic profile of the EO. Figure 2 also shows that other compounds, such as terpinen-4-ol, p-cymene, and D-limonene, are present in lower percentages. Overall, Laurel EO has a rich composition of monoterpenes and oxygenated derivatives, with a particularly high content of 1,8-cineole (eucalyptol).

**Table 1:** Chemical composition and percentage distribution of components of Laurel EO

No	Compound	%	Retention Time	Retention Index
1	Eucalyptol (1,8-cineole)	40.64662	20.0121	1250
2	.α.-Terpinyl acetate	14.51197	40.97225	1759
3	. β.-Phellandrene	9.528318	15.50567	1141
4	1R-α-Pinene	6.804142	11.09745	1020
5	.(-)-β-Pinene	5.462534	14.86408	1125
6	Terpinen-4-ol	3.836411	37.15365	1666
7	p-Cymene	2.885939	22.71653	1316
8	D-Limonene	2.014682	19.43192	1236
9	.α.-Terpineol	1.946922	40.7267	1753
10	. γ.-Terpinene	1.627363	21.73422	1292
11	(+)-4-Carene	1.304396	39.24209	1717
12	Carvacrol	1.142828	54.28797	2082
13	Methyleugenol	1.095882	49.83717	1974
14	Linalool	0.940868	34.35405	1598
15	β-Myrcene	0.883944	17.60233	1191
16	3-Thujene	0.768682	11.26628	1039
17	α-Terpinene	0.755993	18.42197	1211
18	Eugenol	0.618481	53.41317	2061
19	Caryophyllene	0.522419	37.8044	1682
20	Camphene	0.447206	12.93012	1056
21	δ-Terpineol	0.384299	39.80126	1730
22	Caryophyllene oxide	0.339806	50.06125	1979
23	Camphor	0.302422	33.36205	1574
24	Terpinolene	0.292305	23.50853	1335
25	2,3-Dehydro-1,8-cineole	0.24257	18.90085	1223
26	Sabinene hydrate	0.241647	31.051	1518
27	(-)-Myrtenol	0.22857	43.88248	1829
28	(1R)-(-)-Myrtenal	0.222776	38.23453	1692

### Particle size distribution of NEms

The droplet size of the laurel EO-based NEms was measured using a Zetasizer (Malvern Instruments), as shown in Fig. 2. The average particle size (Z-average) of the NEms was 72.36 d.nm, and the polydispersity index (PdI) was 0.289.

### Evaluation of acaricidal activity against *Varroa* mites

The acaricidal effect of laurel (*Laurus nobilis*) EO - based NEms on *V. destructor* was evaluated over a 28-day period, with results shown in Table 2. Mite numbers decreased over time in all treatment groups. The L-100 ppm and L-200 ppm groups exhibited a more pronounced reduction in mites on days 21 and 28, while the L-50 ppm group had a more limited effect. Mite numbers remained relatively stable in the positive control group (Flumethrin strip), whereas an increasing trend was observed in the negative control group. Statistical analysis showed that all interaction parameters; treatment (T), time (Tm), and treatment × time (T×Tm) were significant (*p* < 0.001). This indicates that both concentration and duration of application play a significant role in acaricidal efficacy.

**Table 2:** Effect of Laurel EO - based NEms on *Varroa destructor*

Treatment	0.day	7.day	14.day	21.day	28.day	Overall	T F (*p*)	Tm F (*p*)	T*Tm F (*p*)
L-50 ppm	25.0 ± 1.38^gh^	13.0 ± 1.26^d^	11.4 ± 1.03^cd^	9.8 ± 0.8^b−d^	11.0 ± 0.89^b−d^	14.0 ± 1.22^y^	43.096 (< 0.001)	84.252 (< 0.001)	20.820 (< 0.001)
L-100 ppm	23.0 ± 0.95^e−g^	12.6 ± 0.68^d^	10.8 ± 0.58^b−d^	7.4 ± 0.93^ab^	7.8 ± 0.8^a−c^	12.3 ± 1.20^y^			
L-200 ppm	20.2 ± 2.22^e^	12.8 ± 1.36^d^	13.0 ± 1.18^d^	11.2 ± 1.07^cd^	6.0 ± 0.89^a^	12.6 ± 1.09^y^			
P-Control	20.4 ± 1.86^e^	13.4 ± 0.93^d^	13.0 ± 0.71^d^	10.0 ± 0.71^b−d^	12.2 ± 0.86^d^	13.8 ± 0.84^y^			
N-Control	21.4 ± 0.68^ef^	21.6 ± 1.36^e−g^	24.2 ± 1.07^f−h^	27.0 ± 0.63^h^	27.4 ± 1.71^i^	24.2 ± 0.83^z^			
Time	22.0 ± 0.72^B^	14.7 ± 0.85^A^	14.5 ± 1.08^A^	13.1 ± 1.48^A^	13.4 ± 1.82^A^	15.5 ± 0.63			

**^A; B^: Shows statistically significant differences between different time intervals within the same row; ^Y−Z^: Shows statistically significant differences between different applications within the same row; ^a, b,.,i^: Shows statistically significant differences between different application and time interval interactions. T: treatment, Tm: time, T*Tm: interaction. L: Laurel (*Laurus nobilis*) essential oil-based nanoemulsion; 50, 100, 200 ppm: nanoemulsion concentrations; P-control: positive control (fluemethrin); N-control: negative control

**Fig. 3:**
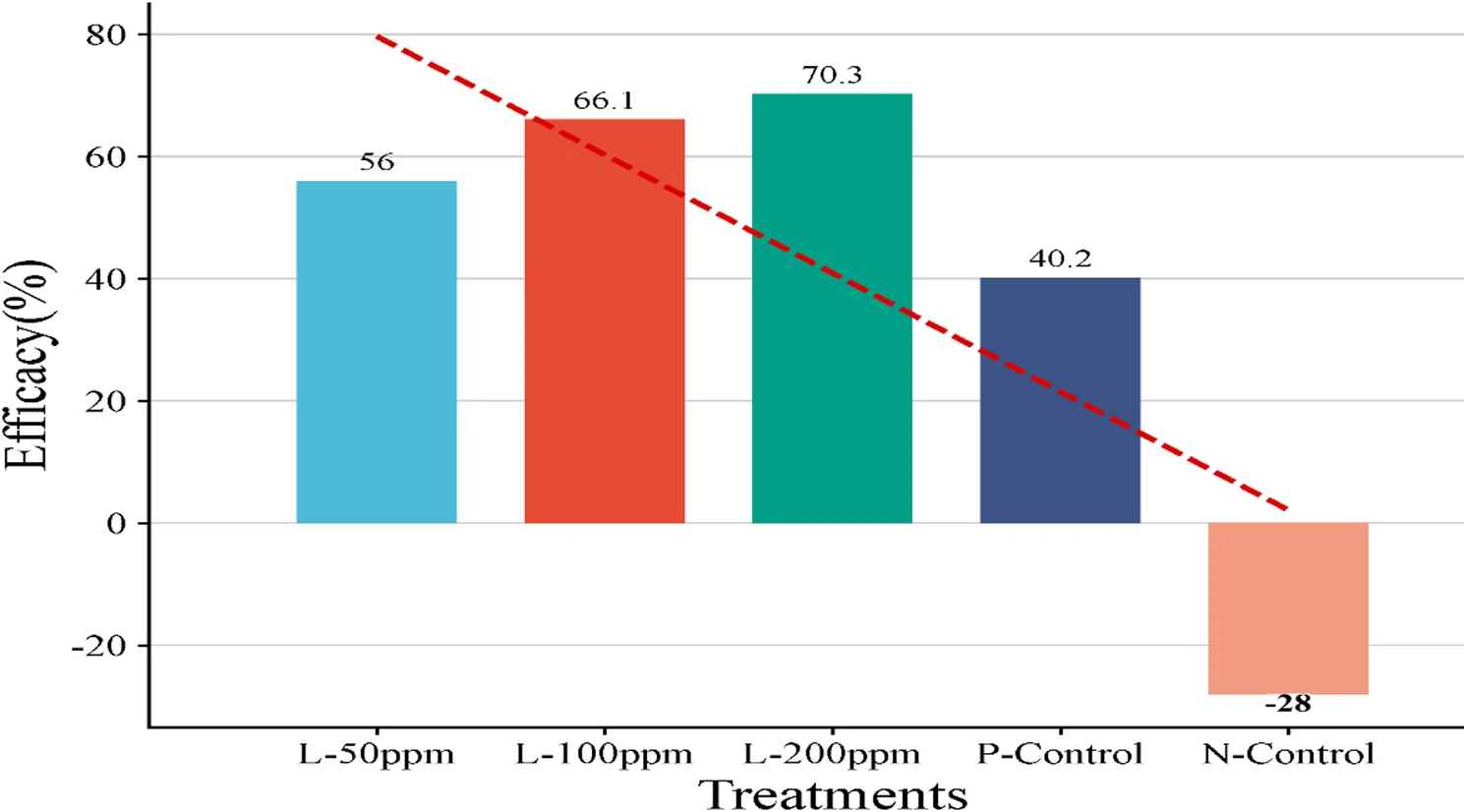
Comparative efficacy of treatments against *Varroa* mites (*L: Laurel essential oil-based nanoemulsion; 50, 100, 200 ppm: nanoemulsion concentrations; P-control: positive control (fluemethrin); N-control: negative control)

The efficacy percentages of the treatments against *V. destructor* are shown in Fig. 3. According to the results, efficacy increased with concentration, with the highest value observed in the L-200 ppm group (70.3%), followed by L-100 ppm (66.1%) and L-50 ppm (56%). The positive control group showed 40.2% efficacy, while the negative control group had a negative value (-28%), indicating an increase in the mite population.

**Fig. 4:**
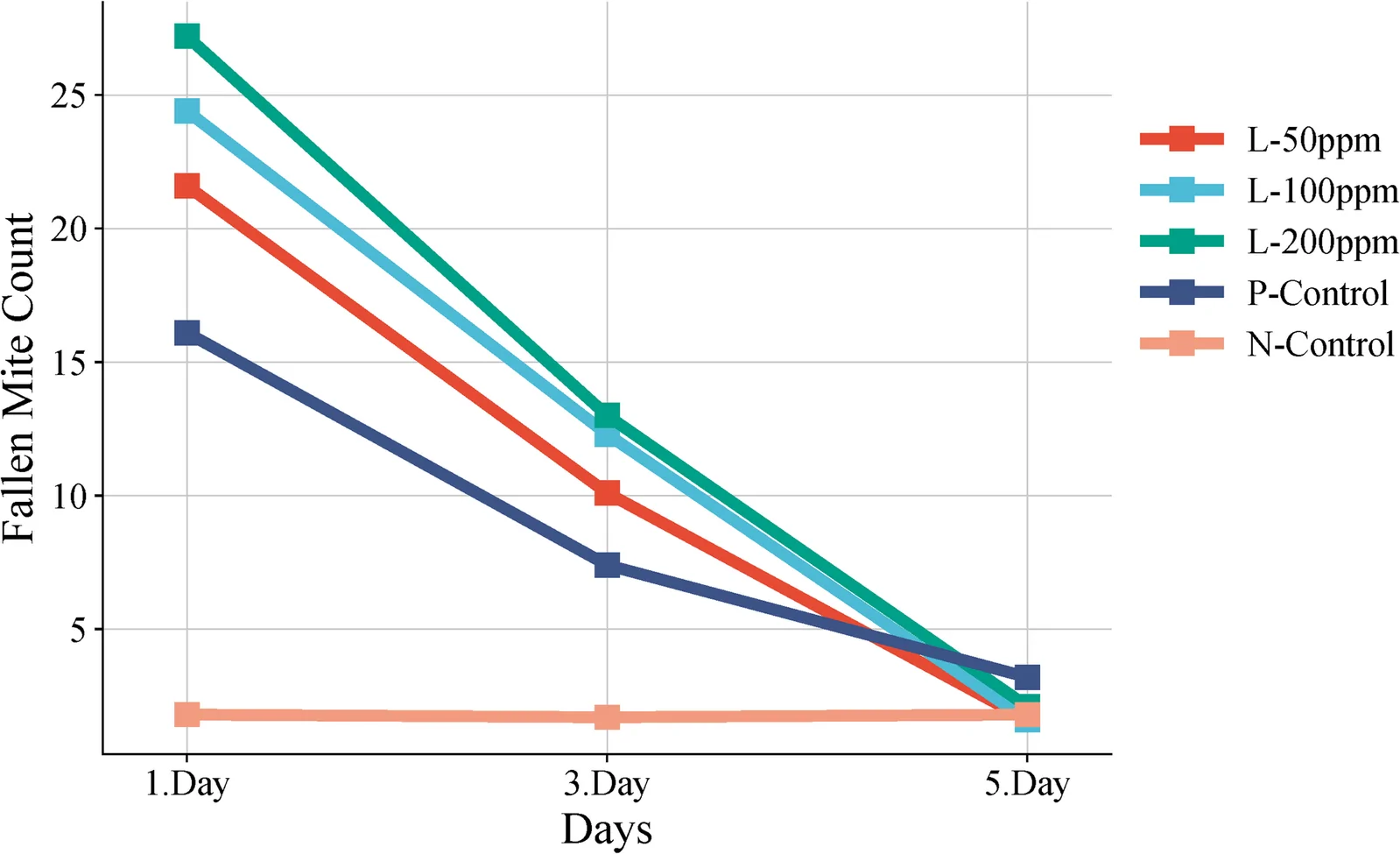
Mean fallen mite counts on days 1, 3 and 5 after treatments (*L: Laurel EO-based NEms; 50, 100, 200 ppm: NEms concentrations; P-control: positive control (fluemethrin); N-control: negative control)

The average number of mites that fell on the 1st, 3rd, and 5th days after application is shown in Fig. 4. A significant decrease in the number of mites falling over time was observed in all NEms groups. The highest values were recorded in the L-200 ppm group on the first day, while the decrease continued in all groups on subsequent days. By the end of the 5th day, the number of mites falling in the NEms -treated groups had decreased to very low levels. Notably, the L-100 ppm and L-200 ppm groups showed a faster and more significant decrease. The decrease was more limited in the positive control group, while values remained low and stable in the negative control group. Overall, NEms applications are effective in the short term after application, and their effect persists over time.

### Results from molecular docking

**Fig. 5:**
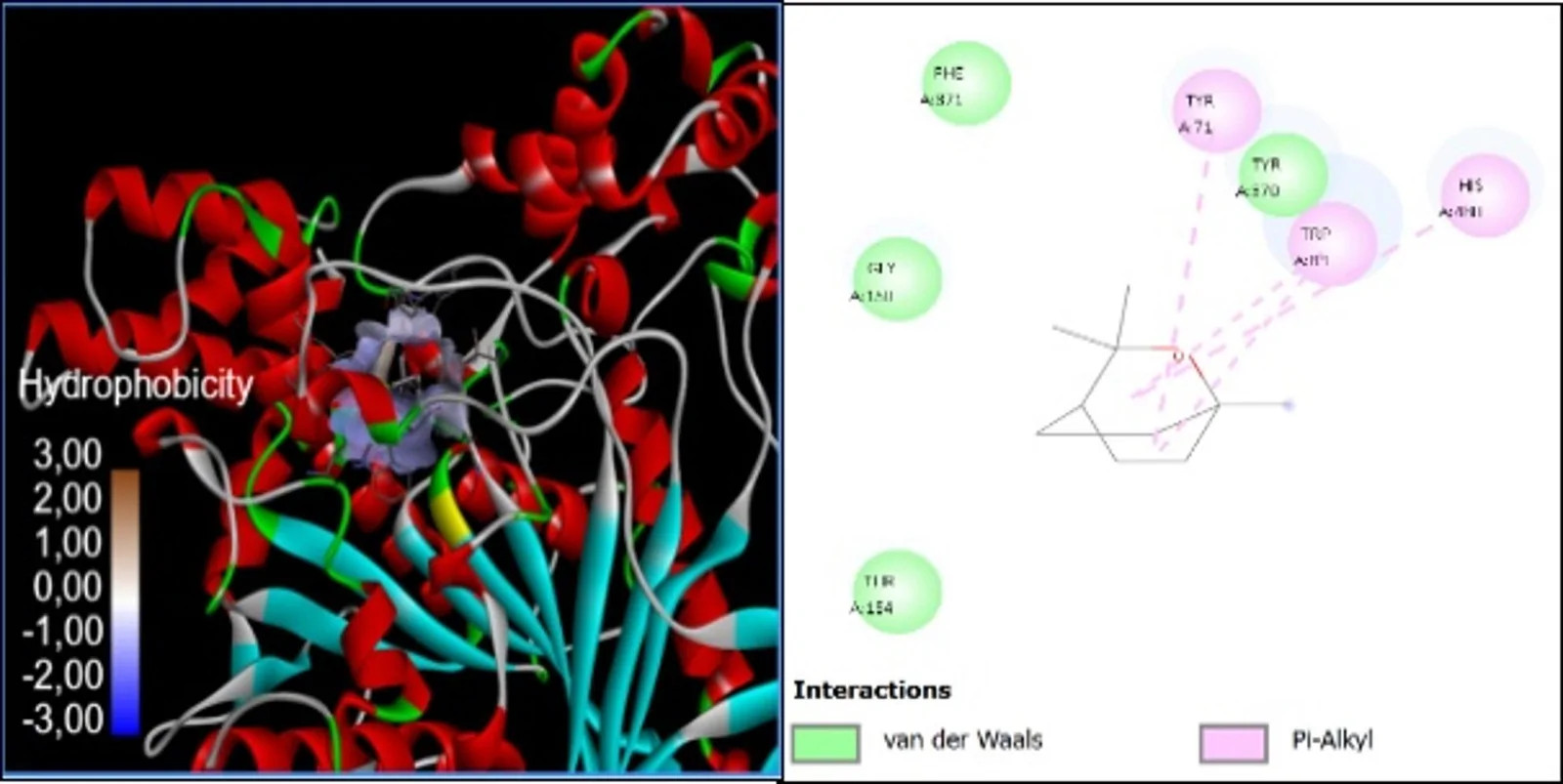
Molecular docking pose of 1,8-cineole within the active site of AChE: 3D representation (left) and 2D interaction diagram (right)

**Fig. 6:**
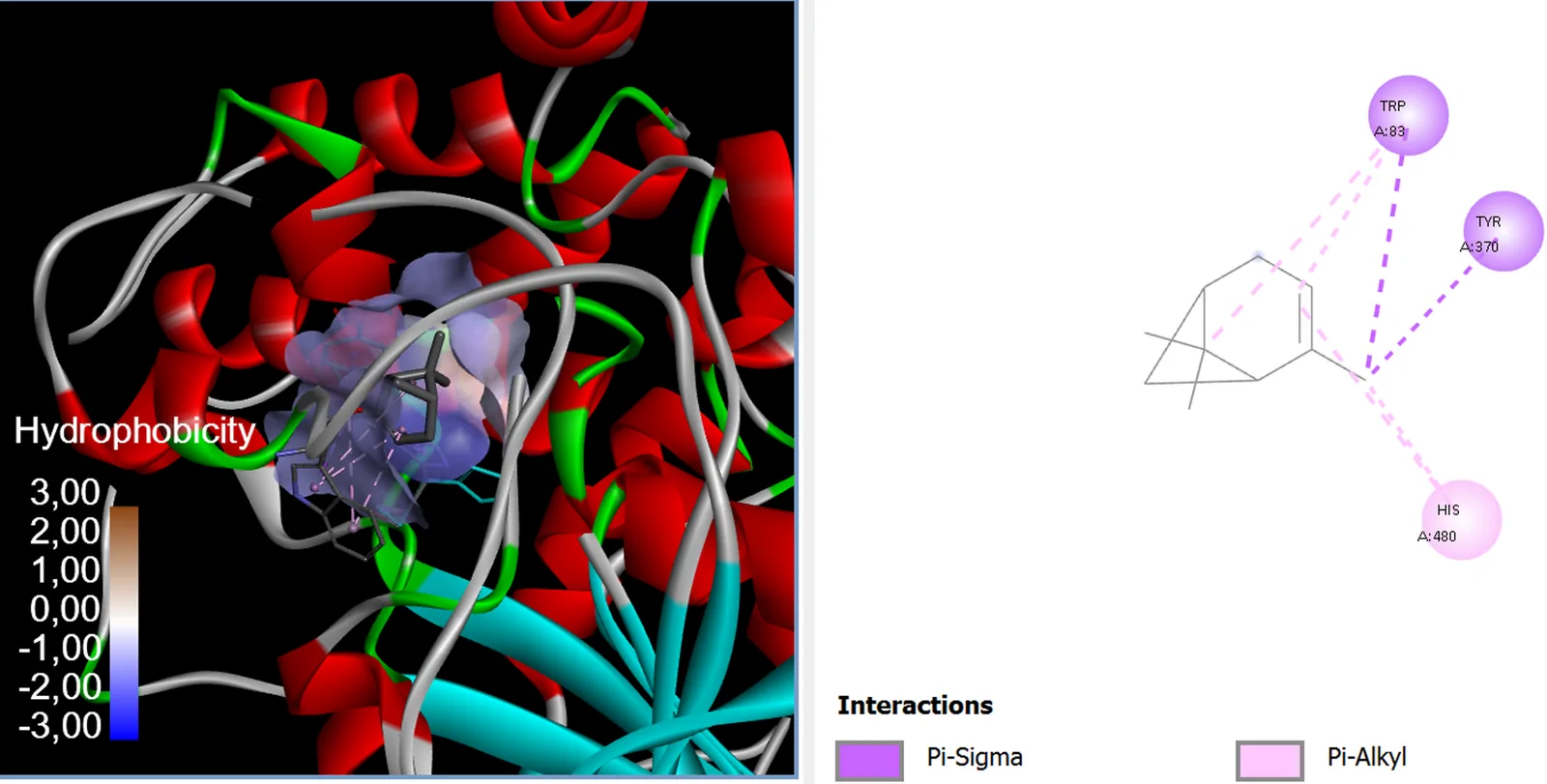
Molecular docking pose of α-pinene within the active site of AChE: 3D representation (left) and 2D interaction diagram (right)

**Fig. 7:**
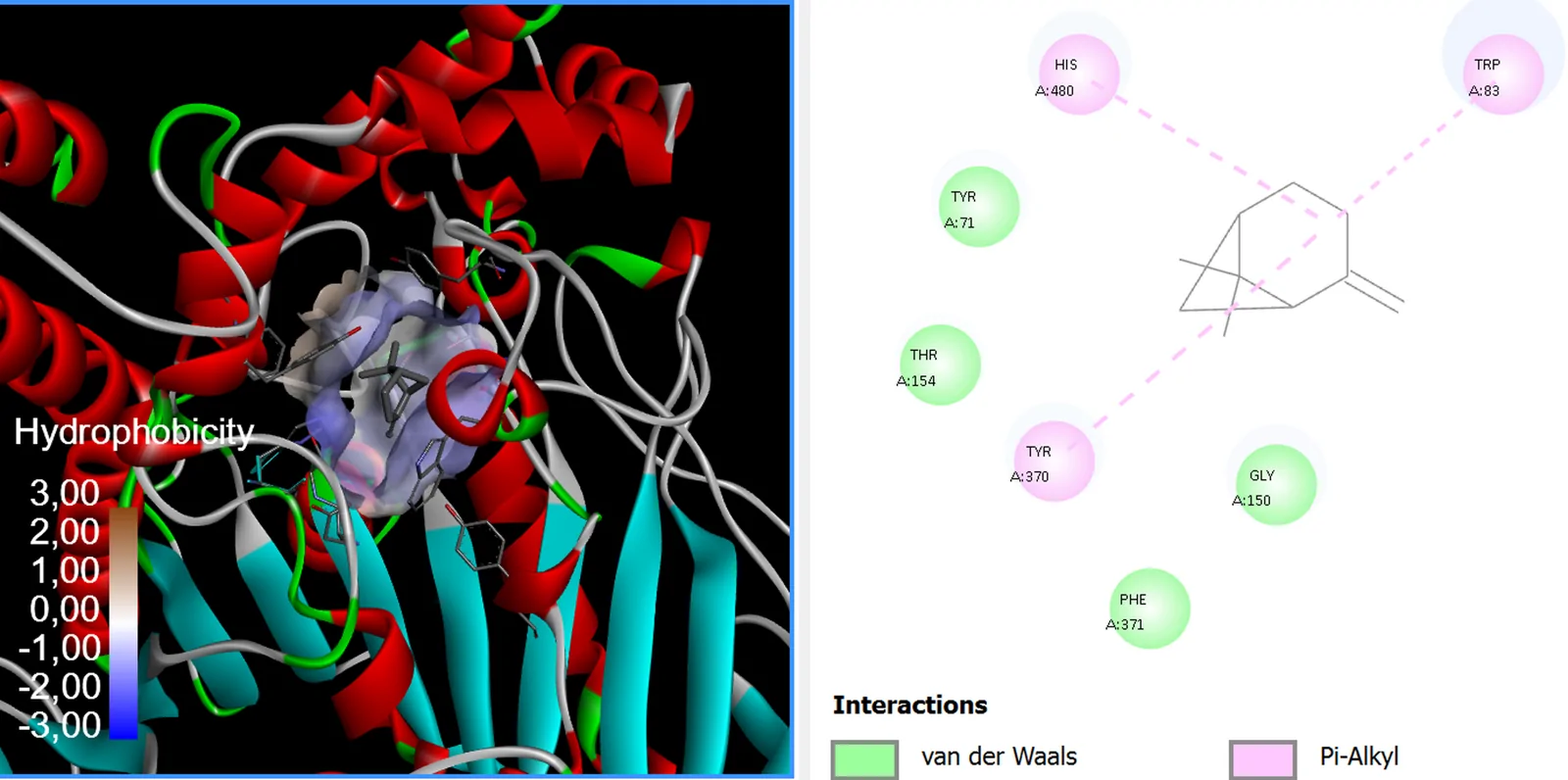
Molecular docking pose of β-pinene within the active site of AChE: 3D representation (left) and 2D interaction diagram (right)

**Fig. 8:**
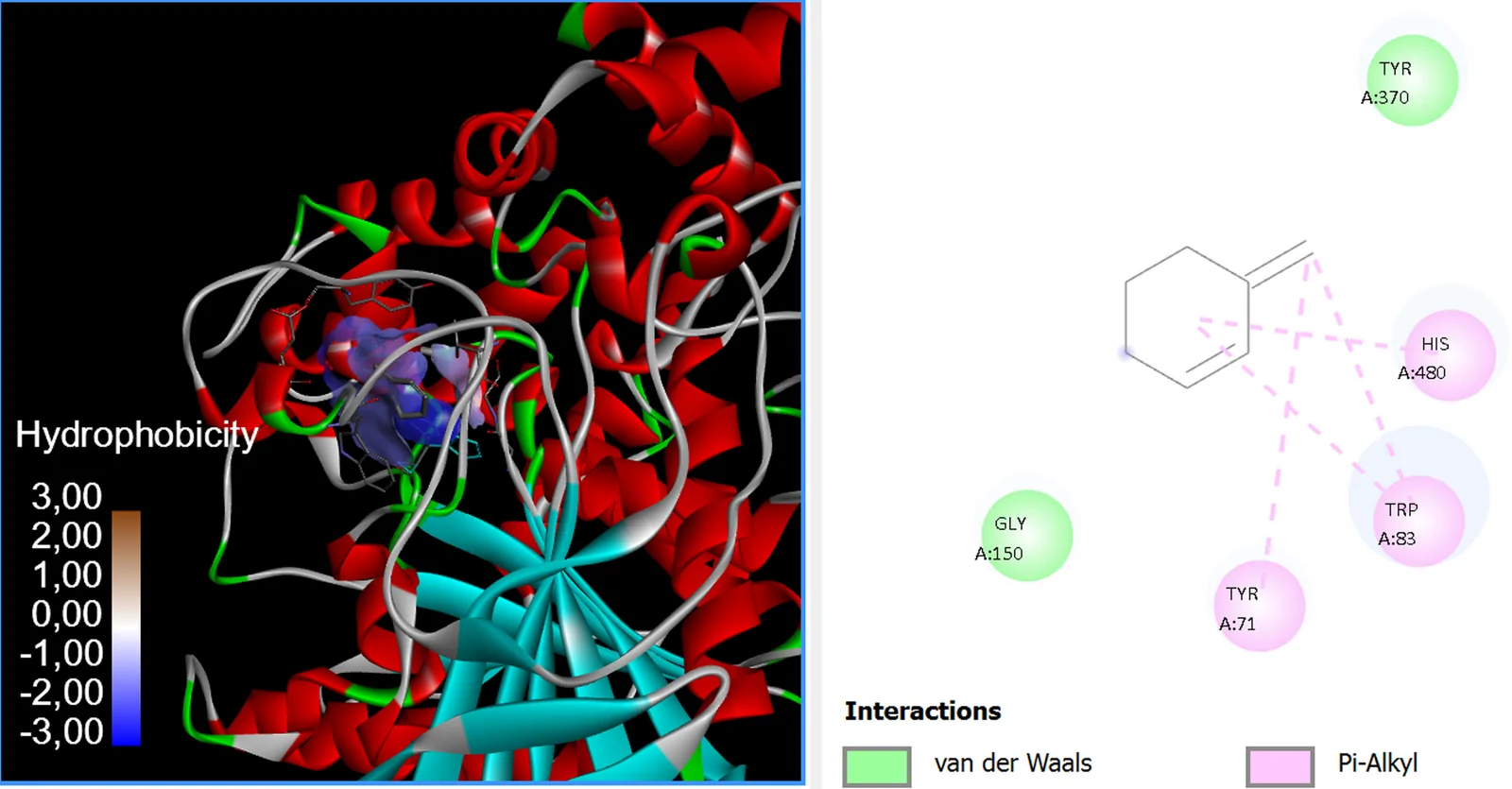
Molecular docking pose of β-phellandrene within the active site of AChE: 3D representation (left) and 2D interaction diagram (right)

**Fig. 9:**
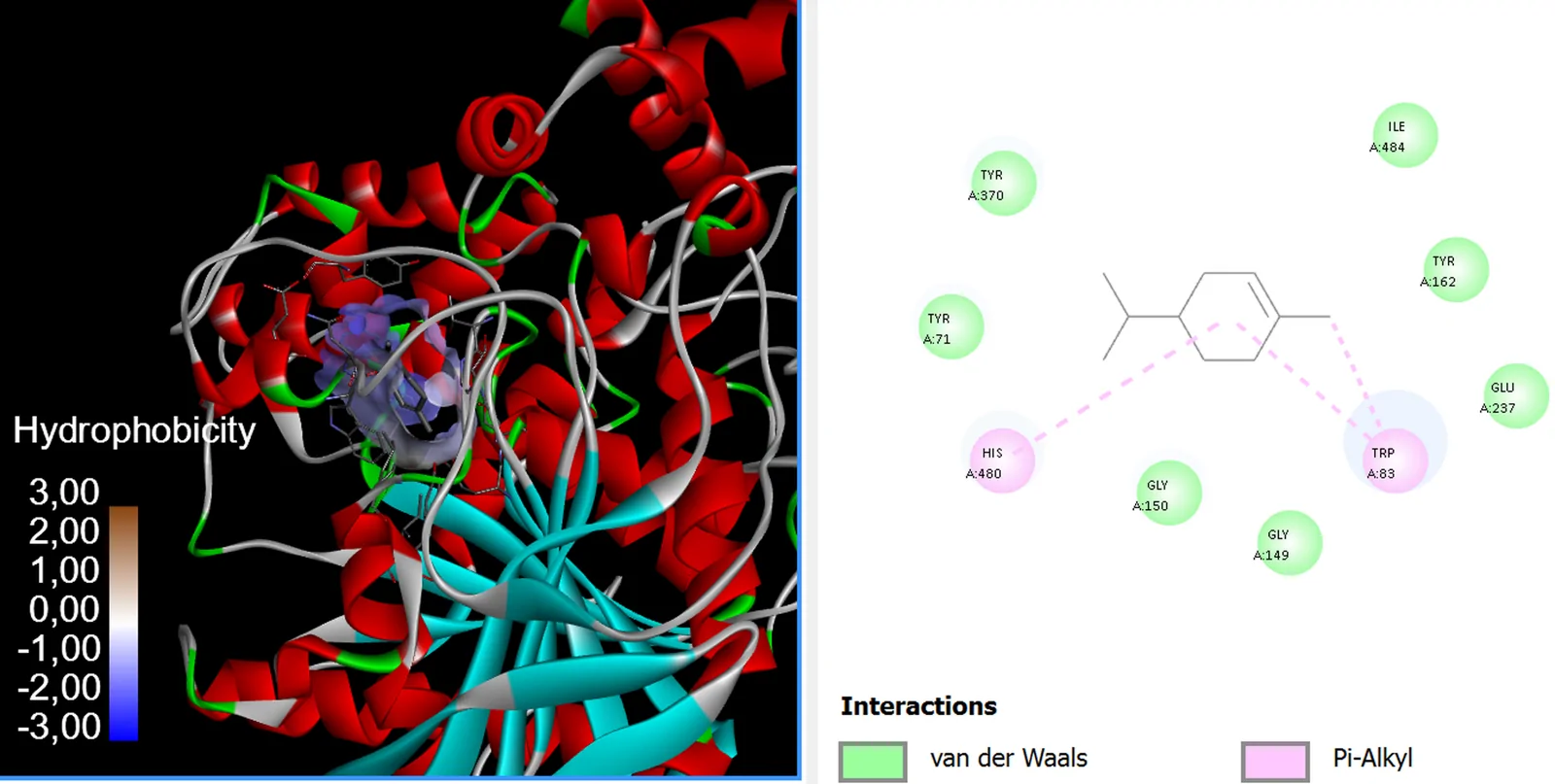
Molecular docking pose of α-terpinene within the active site of AChE: 3D representation (left) and 2D interaction diagram (right)

Molecular docking analysis of the major compounds (1,8-cineole, α-pinene, β-pinene, β-phellandrene, and α-terpinene) identified by GC-MS in bay leaf EO with AChE revealed that all compounds could bind to the enzyme’s active site with varying affinities. According to AutoDock Vina results, α-terpinene showed the highest binding affinity (-6.874 kcal/mol), followed by 1,8-cineole (-6.163 kcal/mol), α-pinene (-5.766 kcal/mol), β-phellandrene (-5.712 kcal/mol), and β-pinene (-5.558 kcal/mol). These values indicate that all tested compounds can form thermodynamically favorable and relatively stable complexes with AChE. Analysis of docking poses (Figs. 5, 6, 7, 8 and 9) showed that the ligands fit well into the active site cavity and are stabilized primarily through hydrophobic interactions and van der Waals forces. Relatively low RMSD values further support the reliability and consistency of the predicted binding modes. In addition, the proximity of these compounds to the enzyme’s catalytic site demonstrates their potential to interfere with enzymatic activity. Taken together, these findings indicate that the main components of bay leaf EO possess considerable AChE inhibitory potential. Among these, α-terpinene and 1,8-cineole appear to interact more strongly with the enzyme, highlighting their potential contribution to the neuroprotective properties associated with this EO.

## Discussion

This field study demonstrates that the developed *Laurus nobilis* EO - NEms exerts a strong acaricidal effect on *Varroa destructor* populations. Furthermore, molecular docking investigations confirm that the main chemical constituents of this botanical oil exhibit significant binding affinities towards the AChE enzyme target. Together, these computational and empirical data strongly suggest that the parasite-killing mechanism of this specific NEms formulation operates directly by disrupting the arthropod cholinergic system.

The parasitic mite *V. destructor* remains the most destructive global threat to the Western honey bee (*Apis mellifera* L.) since its expansion across Europe in the late 20th century. Breeding mite-resistant honey bee lineages is a valuable long-term goal; however, this selection process is highly time-consuming and does not provide immediate relief for the urgent economic needs of the apiculture sector [57]. Consequently, both commercial and hobbyist beekeepers urgently require reliable mite control strategies. Traditional chemical acaricides offer rapid, short-term control, yet these synthetic options have significant operational disadvantages, including high treatment costs, intensive labor requirements, chemical residue accumulation in hive products, and the accelerated development of resistant mite populations [58].

Synthetic acaricides also pose direct toxicological risks to honey bee longevity and human consumer health. In contrast, botanical EOs offer a highly sustainable alternative for integrated pest management. Due to their complex, multi-component chemical profiles, these natural extracts make the development of target-site resistance in mites extremely difficult [59]. EOs consist of volatile aromatic compounds derived from plant tissues and are universally classified as generally recognized as safe substances [60]. The specific extract from *L. nobilis* exhibits potent antibacterial, antifungal, antioxidant, and acaricidal properties [61], while traditionally serving as a therapeutic agent for gastrointestinal disorders [60]. These diverse biological activities are mainly driven by oxygenated monoterpenes, specifically 1,8-cineole, α-terpinyl acetate, and γ-terpinene, which constitute the principal bioactive profile of laurel EO [62].

The chemical composition of EOs can vary according to genetic structure, environmental factors, developmental stage, and extraction methods [42]. The results of this study are consistent with previous research reporting that 1,8-cineole is the main component [60, 63, 64]. However, some studies have found that other components are dominant [65, 66]. These differences are attributed to factors such as plant age, harvest time, plant part used, extraction method, and environmental conditions [60].

Nanotechnology is an innovative approach that enhances biological efficacy by creating systems with sizes ranging from 1 to 100 nm [67]. The average droplet size obtained in this study (72.36 nm) is consistent with the values reported by Özogul et al. [42] and differs from the larger droplet sizes reported in some studies [61]. In NEms systems, droplet size can vary depending on surfactant concentration, oil–water interfacial tension, and the homogenization method used [68]. Furthermore, a low PdI indicates that the system has a homogeneous and stable distribution [60]. These properties can facilitate the transport of active components to the target organism, thereby increasing biological efficacy. The results of this study revealed that laurel oil NEms exhibited a moderate acaricidal effect on *Varroa* mites. This effect is thought to be related to the rich chemical composition of the EO. The literature reports that α-terpinene shows high acaricidal activity against different mite species [69, 70]. Similarly, the effectiveness of 1,8-cineole, the main component of *L. nobilis* EO, against *V. destructor* is supported by various studies [9, 71, 72]. The results obtained at different concentrations in this study are also consistent with the literature [73, 74]. However, the biological activity of EOs may result not only from major components but also from synergistic interactions with minor components.

The low efficacy of flumethrin, used as a positive control, is primarily due to the development of regional resistance in the field. Although historical data report high efficacy rates for flumethrin [75], widespread resistance to synthetic pyrethroids has been extensively documented across Turkey in recent years [76, 77], supporting our study. Mechanistically, this resistance is driven by increased monooxygenase enzyme activity dependent on the cytochrome P450 system in local *Varroa* populations [78]. Therefore, baseline flumethrin efficacy was severely compromised in the tested apiary. This sharp decline in synthetic acaricide susceptibility highlights the critical importance of developing natural product-based alternatives such as laurel NEms.

The literature also supports that NEms formulations enhance insecticidal efficacy. Badawy et al. [79] reported that insecticides in NEms form exhibited higher toxicity and more potent inhibition of AChE activity. Similarly, the results of this study suggest that NEms systems can improve biological efficacy by increasing interaction with target enzymes. Additionally, EOs and their components have been reported to show significant anticholinesterase activity along with favorable pharmacokinetic profiles, strengthening their potential use in the management of neurodegenerative diseases [80]. AChE is a key enzyme responsible for the hydrolysis of acetylcholine in synaptic transmission and is considered an important molecular target for monoterpenes [81]. While AChE inhibition is one of the most commonly investigated mechanisms of action of EOs, these compounds are generally reported to be weak inhibitors [82]. However, cholinergic stimulation induced by AChE inhibitors can lead to acetylcholine accumulation in the synaptic cleft, resulting in serious physiological consequences such as sustained nerve transmission, neuromuscular dysfunction, paralysis, and death. The toxic effects of AChE inhibition are not limited to the cholinergic system but are also associated with oxidative stress mechanisms. Increased free radical production and alterations in the antioxidant defense system heighten the organism’s susceptibility to chemical stressors [83]. Therefore, the effects of EOs on AChE activity play a significant role in their evaluation as potential insecticidal and acaricidal agents [84–86].

In the literature, monoterpenes such as α-pinene, β-pinene, limonene, carvacrol, menthol, and especially 1,8-cineole have been reported to affect insect AChE [81, 84–86]. In this context, the interaction of bay leaf oil components with AChE can be considered an important mechanism explaining the observed biological effect. Molecular docking studies are widely used to reveal the interactions of ligands with residues in the active site of AChE [87]. The AChE enzyme contains different functional regions, such as the catalytic triad (Ser203, Glu337, His447), oxyanion hole (Gly121, Gly122, Ala204), anionic subregion (Trp86, Tyr133, Tyr337, Phe338), and acyl pocket (Phe295, Phe297) [88]. Interactions with these regions play a decisive role in the binding stability and efficacy of inhibitors. Previous studies have shown that Laurel EO has significant anti-AChE activity [89]. Similarly, the binding energies reported by Al-Rajhi et al. [90] (-6.78 and − 6.14 kcal/mol) are consistent with the results obtained in this study. In addition, Jaradat et al. [91] stated that 1,8-cineole was a dominant component in molecular docking analyses. However, in Sugeçti [92] study, the interactions of Laurel EO components with juvenile hormone binding protein were investigated, and it was reported that compounds such as quercetin (-7.3 kcal/mol), α-terpinyl acetate (-6.8 kcal/mol), and α-pinene (-6.5 kcal/mol) showed strong binding affinities. It was also reported that 1,8-cineole interacted with the active site and that these interactions were stabilized by hydrophobic and π-bonds. These findings indicate that laurel oil components can exert their effects not only through neurotoxic targets such as AChE but also through hormone regulatory systems.

Several limitations must be considered when interpreting the results of this study. Firstly, the absence of a separate control group for the carrier components (Tween 80, ethanol, and water) makes it difficult to rule out completely the possible physical effects of these components on mite mortality. Although these substances are generally considered to have low toxicity [93], it should be noted that the observed effect may be attributable not only to the EO components but also to the NEms system as a whole.

Furthermore, the study was conducted in a single apiary over a limited period, and factors such as brood dynamics, climatic variability, and intercolony mite movement (drifting and robbing behaviors) may have affected the observed efficiency [94]. However, it is also known that the sugar shaking method used may, in some cases, underestimate the actual mite density [95].

From an application perspective, EO - NEms offer advantages such as low production costs and the use of plant-derived raw materials [96]. However, the need for repeated application due to the short-term effects of these formulations may increase labor requirements and limit their economic feasibility. Additionally, due to local laboratory infrastructure constraints and resource limitations, comprehensive target-organ toxicity assessments and chemical residue analyses in hive products could not be performed at this preliminary stage. Consequently, the current findings are restricted primarily to immediate acaricidal efficacy and baseline colony tolerability. Future independent trials are strongly recommended to conduct carrier-controlled experiments, perform long-term safety analyses on brood and queen performance, and map precise chemical residue profiles in honey and wax across diverse field conditions.

From a practical perspective, the field applicability and commercial feasibility of the developed NEms are supported by its high efficacy at low concentrations, which directly reduces raw material costs. Mechanistically, the direct liquid spray method enhances biological potency because the nanoscale droplets maximize cuticular contact and facilitate rapid cellular penetration. In terms of resistance management, the complex multi-component chemical profile of natural *Laurus nobilis* EO exerts a multi-target mode of action. This structural complexity greatly limits the ability of *Varroa* populations to develop target-site resistance, offering a sustainable alternative to single-compound synthetic acaricides.

## Conclusion

The developed *Laurus nobilis* EO - NEms offers a reliable and ecologically sustainable tool for controlling *Varroa destructor*. Field trials confirm that this nano-based delivery system achieves significant, dose-dependent acaricidal activity under real apiary conditions. Notably, the formulation outperforms traditional synthetic pyrethroids, which are affected by widespread regional resistance. Molecular docking calculations elucidate the neurotoxic pathways, demonstrating strong structural binding affinities of the major laurel monoterpenes to the arthropod AChE enzyme. Nanoformulation of the botanical matrix directly improves physical stability and ensures immediate cuticular contact during topical spray applications. Although further independent studies are required to assess long-term safety for honey bee brood and queen performance, this multi-component botanical NEms represents a highly effective alternative for integrated pest management. 

## Supplementary Information

Supplementary Material 1.

## Data Availability

Data Availability Statement The data supporting the findings of this study are available from the corresponding author upon reasonable request.

## Abbreviations

EO: Essential oil
NEms: Nanoemulsions
GC-MS: Gas chromatography–mass spectrometry
AChE: Acetylcholinesterase
PDB: Protein Data Bank
PdI: Polydispersity index
T: Treatment
Tm: Time
