# Supplementary material for: Acaricidal activity of Laurus nobilis L. against Varroa destructor: nanoemulsion and molecular docking
Source: BMC Vet Res. 2026 Jun 23;22:517. doi: 10.1186/s12917-026-05669-4 (PMC13548621; doi:10.1186/s12917-026-05669-4)
Supplement: Supplementary file 1 [file 12917_2026_5669_MOESM1_ESM.docx]

**Table S1.** Climatic data of the trial location

| **Climatic Characteristics** | **September** | **October** | **November** | **December** |
| --- | --- | --- | --- | --- |
| **Monthly Average Temperature (°C)** | 20.9 | 13.0 | 5.3 | -0.9 |
| **MonthlyAverage Min. Temperature (°C)** | 14.7 | 7.2 | 1.5 | -3.0 |
| **MonthlyAverage Max. Temperature (°C)** | 28.0 | 20.4 | 9.9 | 1.8 |
| **Average Relative Humidity (%)** | 46.6 | 49.6 | 75.7 | 83.8 |
| **Avg. Precipitation (mm=kg/m²)** | 40.2 | 43.8 | 61.4 | 2.4 |
| **Monthly Average Pressure (hPa)** | 867.6 | 870.9 | 872.2 | 874.7 |

*hPa: Hectopascal, °C: centigrade degree, mm=kg/m²: millimeter=kilogram/square meter, %: percentage
